# Optimal site for fluoroscopic tracer injection for laparoscopic lymphadenectomy

**DOI:** 10.1186/s12916-021-02145-8

**Published:** 2021-10-27

**Authors:** Fernando A. M. Herbella, Marco G. Patti

**Affiliations:** Department of Surgery, Federal University of Sao Paulo, Escola Paulista de Medicina, Rua Diogo de Faria 1087 cj 301, Sao Paulo, 04037-003 Brazil

**Keywords:** gastric cancer, lymphadenectomy, laparoscopic gastrectomy, indocyanine green, fluorescence, submucosal injection, subserosal injection

## Background

The adequate extent of lymphadenectomy in gastric cancer was a controversial topic when Asian authors tried to show benefits of this approach to their Western peers, as the data came mostly from low evidence-based retrospective series. The first randomized clinical trial conducted in the West failed to show benefits, therefore increasing the controversy [1]. These studies were lately criticized, long-term results reviewed, and new trials came out showing real benefits of lymphadenectomy, making gastrectomy with extended lymphadenectomy the standard treatment for gastric cancer today [2]. After the value of lymphadenectomy was accepted, authors focused on how perform it better. Both the laparoscopic and the robotic approach proved to be similar to open surgery in the number of harvested lymph nodes, while providing the advantages of minimally invasive operations [3, 4].

Indocyanine green has been used to guide lymphadenectomy in gastric cancer cases since the beginning of the century, with the purpose of identifying sentinel lymph nodes [5], a tactic that never proved to be very useful. With open surgery, indocyanine was only a vital dye that colored the lymph nodes in green. With minimally invasive surgery, indocyanine became a fluorescent marker that glitters under near-infrared light. This has been shown to enable the retrieval of a higher number of lymph nodes as demonstrated by the same group from China [6], although we have questioned how indocyanine helps: does it illuminate smaller lymph nodes or increase the area of dissection? [7]

## Main text

Chen et al. [8] went beyond the question of the safety and utility of indocyanine green for gastric lymphadenectomy to try to evaluate the best site to inject the dye, either via the submucosal or subserosal route. The authors randomized 259 patients to either have an endoscopy to inject the tracer the day before the operation (submucosal group) or having the tracer injected during laparoscopy 20 minutes before the beginning of the lymphadenectomy (subserosal group). The number of retrieved lymph nodes was not different between groups (49.8 vs. 49.2, p=0.713). This shows that the diffusion of the tracer is the same irrespective of the technique. The drug is probably injected in the same layer, by either technique. It is surprising that the drug injection time was not an influence on the number of retrieved lymph nodes. The authors concluded that submucosal injection is more costly and associated with decreased patient satisfaction, due to the necessity to do an endoscopy before the operation. The authors recommend that the submucosa injection should not be abandoned as it is still important when dealing with small tumors when a preoperative endoscopy is needed to locate and mark the tumor.

## Conclusion

There are still some questions to be answered and we hope this group working extensively on the topic will be able to teach us more. We are curious to know if other markers are better than indocyanine, and if a different marker will be able to distinguish metastatic lymph nodes from normal ones.

## Data Availability

Not applicable
